# Progressie nierschade vertraagt met juiste voedingsadviezen

**DOI:** 10.1007/s12467-022-0636-y

**Published:** 2022-02-22

**Authors:** Merel Hazewindus

**Affiliations:** Bohn Stafleu van Loghum, Houten, Netherlands

Het overgrote deel van de type 2-diabetespatiënten in Nederland wordt behandeld in de eerste lijn, waarbij de diëtist een prominente rol speelt. 47% van de diëtisten geeft aan dat diabetespatiënten hun grootste patiëntengroep vormen, blijkt uit onderzoek van de Nederlandse Vereniging van Diëtisten (NVD).^1^ Minstens de helft van de diabetespatiënten krijgt uiteindelijk te maken met bijkomende complicaties of comorbiditeiten.^2,3^ Dit percentage neemt toe naarmate de diabetespatiënt ouder wordt.^4^ Meer dan driekwart van de diëtisten in het eerdergenoemde NVD-onderzoek geeft aan diabetespatiënten met één of meer comorbiditeiten in hun praktijk te zien. De meest voorkomende diabetesgerelateerde comorbiditeit is diabetische nefropathie of een andere vorm van nierschade.^5,9^ In 2020 zijn de voedingsrichtlijnen voor diabetespatiënten met nierschade herzien. Dit maakt dat de eerstelijns diëtist meer verantwoordelijkheid heeft gekregen voor deze patiënten. Niet alleen is uit onderzoek gebleken dat het voor deze groep erg moeilijk is om een gepersonaliseerd dieet samen te stellen, van een diëtist wordt nu verwacht dat ze zelfstandiger stappen kan ondernemen. De richtlijnen onderstrepen dat samenwerking tussen diëtist en arts belangrijker is geworden. Hierbij is het doel om de diabetespatiënt met nierschade een betere kwaliteit van leven te geven.

## Nierschade en diabetes

Bij circa 12% van de type 2-diabetespatiënten wordt milde tot matige nierschade aangetoond op het moment van diagnose.^5,6,7^ Diabetische nefropathie wordt veroorzaakt door langdurige hyperglykemie. Het is een vorm van diabetesspecifieke nierschade die leidt tot toenemend eiwitverlies en uiteindelijk terminale nierinsufficiëntie. Kenmerkend voor deze aandoening is de hoge uitscheiding van albumine; 60% van het totale eiwitverlies in de urine is albumine. Bij andere nierziekten is dit circa 25%. Diabetische nefropathie gaat bijna altijd gepaard met hypertensie. In sommige gevallen ontwikkelt de diabetespatiënt eerst hypertensie, die later nierschade of diabetische nefropathie veroorzaakt. Het sterfterisico bij deze patiënten neemt aanzienlijk toe als gevolg van hypertensie of andere vormen van hart- en vaatziekten.^2,3,6^

## Gevolgen verlagen of stoppen medicatie

In eerste instantie zal er sprake zijn van nierinsufficiëntie, waarbij de nieren onvoldoende capaciteit hebben om afvalstoffen te filteren en uit te scheiden. Helaas is de prognose van diabetische nefropathie slecht; uiteindelijk zal 80% na 10 jaar nierfalen ontwikkelen.^5,6,8^ Diabetespatiënten met ernstige nierschade of nierfalen hebben een verhoogd risico op hypoglykemieën doordat bloedglucoseverlagende middelen minder goed worden uitgescheiden door de nieren.^8^ Dit resulteert vaak in het verlagen van de medicatie of zelfs het tijdelijk stoppen van de medicatie. Om te voorkomen dat een patiënt verder achteruitgaat, wordt vanaf dat moment de nadruk nog meer op specifieke voedingsinterventies gelegd, zoals het beperken van eiwitten, zout en kalium. Hierdoor kan bij de patiënt het gevoel ontstaan 'niks meer te mogen eten'; minder koolhydraten vanwege de glucose, minder eiwitten vanwege de nieren, minder groente en fruit vanwege het hoge kaliumgehalte.

## Voedingsinterventies die de achteruitgang in nierfunctie vertragen

De voedingsadviezen voor diabetespatiënten met nierschade zijn gebaseerd op de *Richtlijnen Goede Voeding*, waarbij er verschillende voedingsrichtlijnen bruikbaar zijn.^9^ De belangrijkste punten in de meeste richtlijnen voor diabetespatiënten met diabetische nefropathie of nierschade zijn gericht op:

- de glucoseregulatie;
- het voorkomen van overgewicht;
- de consumptie van micronutriënten (natrium, kalium, fosfaat);
- de consumptie van macronutriënten (eiwit, vetten);
- vochtbeperking;
- alcoholgebruik.

Bovenstaande punten zijn enerzijds gekoppeld aan bloedglucoseregulatie en anderzijds aan de preventie van verergering van nierschade.^8^

## Preventie van verergering van nierschade

De richtlijnen van de *Kidney Disease: Improving Global Outcomes* (KDIGO, 2020) zijn gericht op patiënten met nieraandoeningen, waarbij er ook richting gegeven wordt aan diabetespatiënten met nierschade. Samenwerking tussen diëtist en arts blijft hierbij belangrijk; een gezondere levensstijl kan door een diëtist gemonitord worden en farmacologisch management wordt door de arts uitgevoerd. Bovendien kunnen beide partijen de patiënt ondersteunen bij preventief zelfmanagement.

Belangrijke KDIGO-uitgangspunten zijn een gepersonaliseerd dieet (groenten, fruit, granen, vezels, plantaardige eiwitten, onverzadigde vetten en noten) met een zoutbeperking van < 2 gram/dag. Daarbij hanteert deze richtlijn een eiwit-inname van 0,8 gram eiwit/kg lichaamsgewicht/dag wanneer er geen sprake is van dialyse. Dit wordt verhoogd naar 1 tot 1,2 gram eiwit/kg lichaamsgewicht/dag bij patiënten die wel gedialyseerd worden. Tevens wordt deze groep patiënten aangeraden om minimaal 150 minuten per week te sporten. 

## Richtlijnen in tegenspraak

In de praktijk blijkt het erg moeilijk om een diabetespatiënt met nierschade te behandelen met voedingsinterventies. Voor nierpatiënten gelden namelijk andere voedingsrichtlijnen dan voor diabetespatiënten, en deze richtlijnen zijn soms in tegenspraak met elkaar. Zo kan het zijn dat de voeding die een nierpatiënt voorgeschreven krijgt een schadelijk effect heeft op het behouden van de juiste bloedglucosewaarden en vice versa. Omdat de bloedglucoseregulatie belangrijker wordt geacht dan de nierschade, is het advies aan diëtisten zich te richten op de voeding die een bloedglucoseregulerende werking heeft. Het gevolg is dat veel diëtisten zich conform de richtlijnen dan toch richten op de bloedglucose en niet op het voorkomen of verminderen van nierschade, omdat het simpelweg bijna onmogelijk is om dat met voeding te doen.

## Voedingsdilemma's bij dialyse

Het wordt nog moeilijker wanneer de diabetespatiënt gedialyseerd moet worden. Nierdialyse heeft een directe impact op de bloedglucoseconcentraties doordat glucose en ook insuline worden uitgescheiden tijdens de dialyse. Als gevolg hiervan moet het dieet worden aangepast. Zo is het bij nierschade essentieel de inname van kalium te verminderen, maar dit is vrijwel niet verenigbaar met de richtlijnen voor diabetespatiënten. Als de richtlijnen voor groente en fruit, peulvruchten en noten worden opgevolgd, gaat de inname van kalium namelijk verder omhoog. Zo kan een overschot aan kalium uiteindelijk leiden tot een hartstilstand. Kalium blijkt dan ook voor diabetespatiënten met nierschade de beperkende factor te zijn bij het naleven van de voedingsrichtlijnen en vormt dus een grote uitdaging voor de diëtist.^5,8,9^ Daarbij komt dat het gebruik van extra medicatie tegen eventuele andere aandoeningen de voeding nog meer aan banden kan leggen. Zo zijn er bepaalde medicijnen tegen hypertensie die de concentraties kalium in het bloed verhogen. Ondanks het feit dat die medicatie hypertensie tegengaat, is dit ongunstig voor de nieren.

## Multidisciplinaire samenwerking

Praktische handvatten om nierschade zo veel mogelijk te beperken en de glucoseregulatie te handhaven, worden gegeven in de vernieuwde *NDF-voedingsrichtlijn Diabetes (2020)*.^5^ De NDF geeft daarnaast praktisch advies over ziektespecifieke drinkvoeding die bij sommige diabetespatiënten een uitkomst kan bieden, zoals bij patiënten met nierschade. De nieuwe adviezen van de NDF voor deze patiëntengroep zijn werkbaar, maar wel wordt er meer geëist van de eerstelijns diëtist. Een diëtist moet niet alleen de juiste voedingsinterventie kunnen toepassen, maar moet ook meer kennis vergaren over de aandoening(en) en medicijnen, wetenschappelijke artikelen lezen, extra opleiding volgen en soms lastige beslissingen nemen. De verschillende voedingsuitdagingen vragen om een multidisciplinaire aanpak. Eén van de belangrijkste punten in de vernieuwde *NDF-voedingsrichtlijn Diabetes* is dan ook dat een multidisciplinaire aanpak het risico op achteruitgang en het ontstaan van comorbiditeiten bij diabetespatiënten kan voorkomen. De belangrijkste taak van de diëtist is om preventief te werken en daarbij te voorkomen dat de aandoening verslechtert. Indien een patiënt pas wordt doorgestuurd wanneer er al sprake is van een (sterke) achteruitgang en/of comorbiditeiten zoals nefropathie, wordt het werk van de diëtist een ware uitdaging.

## Toename voedingsdilemma's

De voedingsdilemma's nemen toe wanneer de nierschade bij de diabetespatiënt verergert en wanneer er sprake is van meerdere aandoeningen tegelijk. Omdat de prognose van deze patiëntengroep slecht is, wordt het ook steeds moeilijker om een volwaardig dieet te kunnen samenstellen. Naarmate de nierschade verergert bij een diabetespatiënt zal er ook rekening gehouden moeten worden met verminderde eetlust en veranderende smaak. Een goede voedingsinterventie is in dergelijke gevallen uiterst belangrijk, maar ook bijzonder moeilijk. Uiteindelijk moet de diëtist een voedingskeuze maken die misschien niet meer in overeenstemming is met de dieetrichtlijnen. 

Een ander dilemma doet zich voor bij de groeiende groep ouderen met diabetes en diabetische nefropathie. Deze kwetsbare groep heeft een korte levensverwachting.^2,8^ Uit onderzoek is gebleken dat intensieve bloedglucoseregulerende behandelingen in deze groep niet zinvol zijn; er kan vaak alleen nog gekeken worden naar wat haalbaar is en waar de prioriteiten moeten liggen.

## Ouderen met comorbiditeiten

Uit de meeste voedingsrichtlijnen komt het belang van een persoonsgerichte aanpak naar voren. Het lastige is dat er geen eenduidige definitie bestaat van een diabetespatiënt met nierschade. Zeker niet voor patiënten met bijkomende aandoeningen. Uit buitenlandse bronnen blijkt dat voedingsrichtlijnen vaak tekortschieten bij diabetische nefropathie en zeker in combinatie met andere comorbiditeiten.^10,11^ Het risico op ondervoeding in deze patiëntengroep is dan ook relatief groot wanneer er bepaalde beperkingen gelden voor de ene aandoening die interfereren met de andere aandoening. Dit laat ook het voorbeeld van kaliumreductie zien.^1,2^

## Mogelijke oplossingsrichtingen 

Eén van de oplossingen is het geven van ziektespecifieke voeding, zoals een drinkvoeding. In een onderzoek met 30 patiënten kregen de patiënten allen een gepersonaliseerd dieet voorgeschreven, waarbij in sommige gevallen de diëtist drinkvoeding adviseerde. Uit de resultaten van de kwalitatieve analyse bleek dat bij een groot deel van de patiënten die drinkvoeding kregen een verbetering dan wel een stabilisatie van bloedwaarden zichtbaar werd. Hoe ouder de patiënt, des te moeilijker het bleek om de nierfunctie stabiel te houden. Met name bij patiënten > 70 jaar bleek de nierfunctie minder goed te verbeteren dan bij jongere patiënten na het starten van de voedingsinterventie. Hoewel niet alle patiënten dezelfde meetmomenten hadden, lieten de eGFR-resultaten zien dat met name mannen > 70 jaar kwetsbaarder zijn dan vrouwen van dezelfde leeftijd. In de leeftijdscategorie 60-69 jaar bleken vrouwen moeilijker te stabiliseren. Zie voor de onderzoeksopzet en de resultaten: https://www.abbottnutritionprofessionals.nl/patientenonderzoek

## Meerwaarde van drinkvoeding

De huidige NDF-richtlijn adviseert drinkvoeding pas in te zetten wanneer iemand niet meer in staat is om met normale voedingsmiddelen in zijn voedingsbehoefte te voorzien.^6^ Een cruciale rol is weggelegd voor de diëtist om dit moment in te schatten en kwantitatief en kwalitatief te onderbouwen. Wanneer drinkvoeding werd ingezet, lieten de resultaten van de onderzochte groep patiënten kwantitatieve en kwalitatieve gezondheidsverbeteringen zien. Geïnterviewde patiënten gaven over het algemeen aan zich beter te voelen wanneer ze drinkvoeding namen.

## Conclusie

Een substantieel deel van de diabetespatiënten krijgt te maken met nierschade, waarbij voedingsinterventies een belangrijke rol spelen om de achteruitgang in nierfunctie te vertragen. Het lastige is dat de voedingsrichtlijnen voor diabetespatiënten met nierschade moeilijk te verenigen zijn gezien de beperkingen in meerdere voedingsgroepen. Het verlagen van eiwitten in de voeding verlaagt het risico op verdere achteruitgang van de nieren. Kalium uit groente en fruit verlagen de bloeddruk en hebben een vochtafdrijvend effect. Indien er sprake is van diabetes en nierschade kunnen bepaalde eiwitrijke voedingsbronnen, maar ook groente en fruit, niet meer gegeten of minder gegeten worden. Bovendien heeft een diabetespatiënt al een beperking in koolhydraatinname. Hierdoor kan de patiënt het gevoel krijgen niets meer te mogen eten. Naarmate de nierschade verergert bij een diabetespatiënt, zal er ook rekening gehouden moeten worden met verminderde eetlust en veranderde smaak. Een goede voedingsinterventie is in dergelijke gevallen uiterst belangrijk, maar ook bijzonder moeilijk. De nieuwe *NDF-voedingsrichtlijn Diabetes (2020)* heeft invloed op de huidige werkwijze van de diëtist. De diëtist moet meer zelf doen, meer kennis hebben over een aandoening en er zijn meer uitdagingen bij patiënten met comorbiditeiten. De vernieuwde richtlijnen leggen meer druk op de diëtist. Ziektespecifieke voeding, zoals drinkvoeding, kan een hulpmiddel zijn bij deze groep patiënten. De huidige richtlijnen adviseren drinkvoeding pas in te zetten wanneer iemand niet meer in staat is om met normale voedingsmiddelen in zijn voedingsbehoefte te voorzien.^5^ Hierbij is een cruciale rol weggelegd voor de diëtist om dit moment kwantitatief en kwalitatief te onderbouwen en in te schatten. Als artsen tijdig de diabetespatiënten doorsturen − nog voordat deze nierschade krijgen − en goed samenwerken met de diëtisten, kan dit in het voordeel van de diabetespatiënten werken. Dit resulteert in meer kwaliteit van leven, een beter dieet en minder risico's op het krijgen van ernstige nierschade of andere aandoeningen.

*Met dank aan dr. Suhail Khan, diëtist/nutritionist. Gebruikte data zijn de in het ziekenhuis gemeten bloedwaarden van patiënten uit zijn praktijk.

## REFERENTIES

1. Nederlandse Vereniging van Dietisten. https://www.nvdietist.nl.
2. Nielen MM., Poos MJJ. Chronische ziekten en multimorbiditeit; Cijfers & Context. Nivel (https://www.volksgezondheidenzorg.info/onderwerp/chronische-aandoeningen-en-multimorbiditeit).
3. Nielen M. Comorbiditeit bij diabetes mellitus. Nivel. 2019. https://www.nivel.nl/nl/publicatie/comorbiditeit-bij-diabetes-mellitus.
4. Serné E, Mourits P, van Strien M. Nationale Diabetes Registratie: Voor een toekomstbestendige en persoonsgerichte diabeteszorg in Nederland. Ned Tijdschr voor Diabetol. 2020. doi:10.1007/s12467-020-0131-2
5. NDF Voedingsrichtlijn diabetes. 2020. https://diabetesfederatie.nl/ndf-toolkit-persoonsgerichte-diabeteszorg/persoonsgerichte-voedingszorg.
6. Federatie Medisch Specialisten. Chronische nierschade. 2018.
7. Hemmelder MH, van Balen J, Scherpbier N, Schenk PW, Tuut MK, Gansevoort RT. Herziening richtlijnen 'Chronische nierschade.' Ned Tijdschr Geneeskd. 2018.
8. DNN. Chronische nierschade, diabetes en voeding. Dietist nierziekten. 2018.
9. Gezondheidsraad. Richtlijnen goede voeding 2015. Gezondheidsraad. 2015.
10. Watch Health Policy. Covid-19. 2020. https://healthpolicy-watch.news.WHO. Ensuring people centred diabetes care during the Covid-19 pandemic. https://www.euro.who.int/__data/assets/pdf_file/0011/444791/Diabetes-care-during-COVID-19-eng.pdf
11. Wexler DJ, Grant RW, Wittenberg E, et al. Correlates of health-related quality of life in type 2 diabetes. Diabetologia. 2006. doi:10.1007/s00125-006-0249-9

